# RBM15 promotes COAD progression by regulating the m6A modification of TMC5

**DOI:** 10.1186/s41065-025-00530-4

**Published:** 2025-08-29

**Authors:** Errong Tian, Li Gao, Lan Wu, Limin Qin

**Affiliations:** 1 Department of Pain, Affiliated Hospital of Inner Mongolia Medical University, Hohhot, 010010 Inner Mongolia China; 2 Department of Anesthesiology, Affiliated Hospital of Inner Mongolia Medical University, No.1 North Street, Huimin District, Hohhot, 010010 Inner Mongolia Autonomous Region China

**Keywords:** TMC5, RBM15, COAD, Proliferation, Ferroptosis

## Abstract

**Background:**

Colon adenocarcinoma (COAD) is a frequent digestive system malignancy with high mortality and poor prognosis. Transmembrane Channel-like 5 (TMC5) has been reported to play an oncological role in various cancers. However, the role and mechanism of TMC5 in COAD remain unclear.

**Methods:**

TIMER and UALCAN databases analyzed the expression of TMC5 in COAD. TMC5, RNA-binding motif protein-15 (RBM15), E-cadherin, N-cadherin, Vimentin, Fibronectin, and RAD51 protein levels were determined using western blot. TMC5, RBM15, Ferritin heavy chain 1 (FTH1), and cystine/glutamate antiporter SLC7A11 (also commonly known as xCT) mRNA levels were examined using real-time quantitative polymerase chain reaction (RT-qPCR). Cell proliferation, apoptosis, migration, and invasion were assessed using 5-ethynyl-2’-deoxyuridine (EdU), flow cytometry, and transwell assays. Caspase 3 activity, ROS level, Fe^+^ level, and glycolysis level were detected using commercial kits. Immunofluorescence assay analyzed 53BP1 and γH2AX foci. Role of TMC5 on COAD tumor growth was examined using xenograft tumor model *in vivo.* After SRAMP database analysis, interaction between RBM15 and TMC5 was verified using methylated RNA immunoprecipitation (MeRIP) and dual-luciferase reporter assay.

**Results:**

TMC5 and RBM15 levels were significantly increased in COAD tissues and cells. Moreover, TMC5 silencing could inhibit COAD cell proliferation, migration, invasion, EMT, glycolysis, and induce apoptosis and ferroptosis in vitro, as well as repress tumor growth in vivo. At the molecular level, RBM15 could sustain RNA stability and TMC5 expression through regulating the m6Amodification.

**Conclusion:**

RBM15 could facilitate COAD cell malignant behaviors at least by regulating the stability of TMC5 mRNA, providing a powerful and hopeful target for COAD treatment.

**Supplementary Information:**

The online version contains supplementary material available at 10.1186/s41065-025-00530-4.

## Graphical Abstract

m6A methylation regulator RBM15-mediated upregulation of TMC5 mRNA stability could promote COAD cell proliferation, migration, invasion, and block apoptosis, oxidative stress, and ferroptosis in vitro.

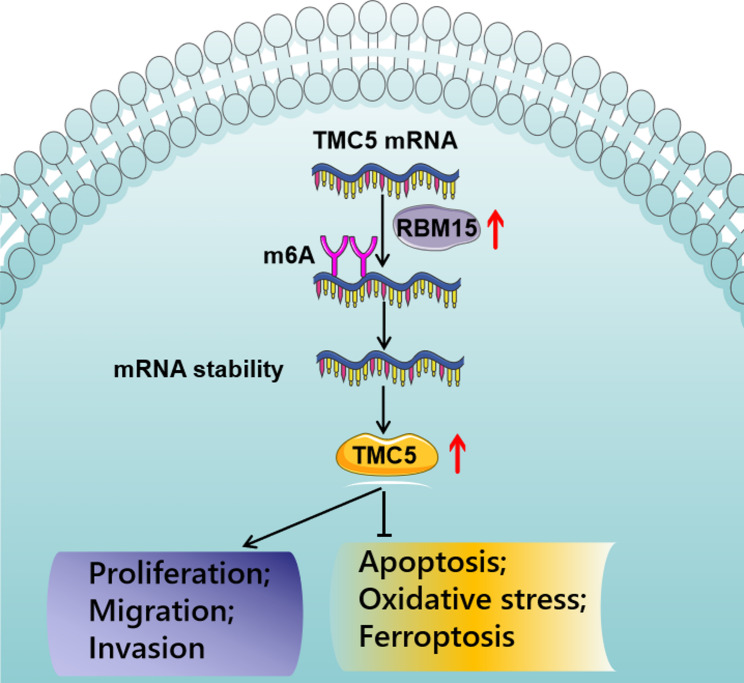

## Introduction

Colorectal cancer remains a highly threatening malignant tumor, with both its incidence and mortality rates on the rise worldwide [1]. Colon adenocarcinoma (COAD) is the main histological subtype of colon cancer, with a significantly higher incidence and mortality risk than rectal cancer [2]. COAD has been recognized as a significant public health challenge and economic burden worldwide [3, 4]. Due to atypical early symptoms, the majority of COAD patients are often diagnosed at an advanced stage with regional lymph node and distant metastasis [5]. Although available therapeutic options, such as surgical resection, adjuvant or neoadjuvant chemotherapy, and radiotherapy, have made remarkable strides, the overall prognosis for patients remains unfavorable [6, 7]. In fact, patients with COAD exhibit significant heterogeneity in overall survival. Many view tumor, lymph node, metastasis (TNM) staging as a key factor in evaluating treatment decisions, but it is insufficient to reveal the biological heterogeneity of COAD [8, 9]. Furthermore, it has been reported that the development of COAD is affected by gene signature and clinicopathological features [10, 11]. Therefore, understanding the underlying mechanisms responsible for COAD and developing novel signatures or biomarkers is imperative for improving COAD treatment.

With the development of next-generation sequencing, several previous investigations have successively discovered regulatory genes participating in the pathogenesis of COAD [12–14]. In mammals, the transmembrane channel-like (TMC) family, consisting of eight members (TMC1-TMC8), possesses about ten transmembrane domains and a conserved TMC domain [15]. Numerous studies have suggested that members of the TMC family play critical roles in the development and carcinogenesis of diverse human tumors [16, 17]. For example, an earlier study has shown that TMC6 and TMC8 mutations are correlated with skin cancer [18]. Furthermore, TMC7 was identified as a potential prognostic biomarker for pancreatic cancer and promotes tumor cell invasion and migration [19]. Notably, a comprehensive bioinformatics analysis has indicated that TMC5 expression was significantly increased in most cancer types and functions as a potential marker for cancer survival [20]. Beyond that, it has been reported that overexpressing TMC5 boosts prostate cancer cell proliferation and cell cycle regulation [21]. Meanwhile, TMC5 was overexpressed in cancerous epithelial cells and might facilitate the development of pancreatic adenocarcinoma by regulating STAT3 ^22^. Of interest, a recent report suggested that TMC5 was obviously dysregulated differential proteins in colorectal cancer [22] but its role and mechanism in COAD are still unknown.

Currently, increasing evidence has indicated that genomic and epigenetic modifications play a vital role in tumorigenesis and development [23, 24]. Among them, N6-methyadenosine (m6A) is a prevalent principal mRNA modification, and is added to mRNA or non-coding RNAs by methyltransferases (“writers”), containing METTL3/14, WTAP, VIRMA, and RNA-binding motif protein-15 (RBM15) [25]. On the contrary, the erasers (FTO and ALKBH5) remove the RNA methylation mark [26]. Meanwhile, the readers, such as IGF2BP1/2/3 and YTHDF1/2/3, are able to control RNA metabolism by recognizing and binding to bases modified by m6A [27]. As an important modulatory mechanism for post-transcriptional gene expression control, m6A modification is implicated in RNA stability, cleavage, decay, translation, and nuclear export [28]. Interestingly, RNA-seq transcriptome data have identified that RBM15, a key regulatory factor in m6A methylation, was apparently upregulated in COAD patients [29]. Furthermore, it has been reported that RBM15-mediated m6A modification of MyD88 mRNA sustains MyD88 stability and accelerates the proliferative and invasive abilities of colorectal cancer cells [30]. Apart from that, overexpressing RBM15 could expedite colorectal cancer cell growth and migration by increasing KLF1 stability [31]. In this research, the online tool SRAMP database found the possible m6A site of TMC5 mRNA. Further analysis identified the binding between RBM15 and TMC5 in COAD cells. Hence, we aimed to investigate whether RBM15 could stimulate COAD progression by controlling TMC5 mRNA stability.

## Materials and methods

### Clinical samples and cell culture

With the approval of the Ethics Committee of Affiliated Hospital of Inner Mongolia Medical University, Sixty-nine fresh COAD specimens and matched paracancerous tissues were collected. All the participants gave written informed consent. Immediately after surgery, these tissues were stored at -80˚C until used.

In an incubator at 37˚C with 5% CO_2_, COAD cell lines (Caco-2, CL-0050; SW620, CL-0225; LoVo, CL-0144; Procell, Wuhan, China) were maintained in corresponding media (CM-0050, CM-0225, CM-0144; Procell). Besides, a normal human colon mucosal epithelial cell line (NCM460, SNL-519, SUNNCELL, Wuhan, China) was cultured in specific medium (SNLM-519, SUNNCELL).

### Western blot assay

Total tissue and cells were lysed with RIPA buffer (CST, Beverly, MA, USA) containing protease and phosphatase inhibitors. After being quantified with BCA method, samples of cell lysates were denatured at 100˚C for 5 min in loading buffer. Subsequently, protein samples were resolved with SDS-PAGE gel and transferred onto nitrocellulose membranes (Millipore, Molsheim, France). After blocking, the membranes were incubated overnight at 4˚C with appropriate diluent primary antibodies: TMC5 (PA5-95696, Invitrogen, Paisley Scotland, UK), RBM15 (PA5-99993, Invitrogen), E-cadherin (ab76055, 1:1000, Abcam, Cambridge, MA, USA), N-cadherin (ab76011, 1:5000, Abcam), Vimentin (ab20346, 1:1000, Abcam), Fibronectin (ab2413, 1:1000, Abcam), RAD51 (ab133534, 1:10000, Abcam), and β-actin (PA5-85271, Invitrogen; ab8226, Abcam), followed by incubation with secondary antibody. Finally, protein signals were visualized by ECL kit (Solarbio, Shanghai, China).

### RT-qPCR

For mRNA studies, the purification of total RNAs from clinical samples and cell lines was performed using Trizol reagent (Invitrogen). After synthesizing template DNA with BeyoRT II M-MLV reverse transcriptase (Beyotime, Shanghai, China), amplification reaction was carried out with TB Green Fast qPCR Mix (Takara, Dalian, China) on Thermal Cycler CFX6 System (Bio-Rad, Hercules, California, USA). At last, target gene expression was assessed with the 2^–ΔΔCt^ approach, using β-actin as internal control. Primers used were shown in Table 1.

**Table 1:** Primers sequences used for PCR

Name		Primers for PCR (5’-3’)
RBM15	Forward	CGAGATAGGAAGCACCGGAC
	Reverse	CCCCATCCTGTTTCTGGGAC
TMC5	Forward	TTTTCCCATCCTTCACCGGG
	Reverse	CAGGCCGTGTACTTAGGGTG
FTH1	Forward	GCTCTACGCCTCCTACGTTT
	Reverse	CCTGAAGGAAGATTCGGCCA
SLC7A11	Forward	ATCCATGGCCATTGTCACCA
	Reverse	CCCAGTAGCCGCTCAGAAAA
β-actin	Forward	CCGCGAGAAGATGACCCAG
	Reverse	GATAGCACAGCCTGGATAGCA

For the RNA stability assay, 1 × 10^5^ Caco-2 and LoVo cells were plated into 6-well plates. Up to 60% confluency after 24 h, cells were exposed to actinomycin D (a transcriptional inhibitor, Sigma-Aldrich, St. Louis, MO, USA) for 0, 3, 6, or 9 h. Finally, RT-qPCR assay was used to analyze the half-life of TMC5 mRNA.

### Cell transfection

First of all, the lentiviral pLKO.1 constructs encoding shRNAs against human TMC5 (sh-TMC5) or human RBM15 (sh-RBM15), and pLKO.1 constructs encoding human RBM15 cDNA (NM_022768.5), or TMC5 cDNA (NM_001105248.1) were obtained from Genechem (Shanghai, China). Subsequently, HEK293T cells (CL-0005, Procell) at 80% confluency were transfected with lentivirus package plasmids and these constructs using polyethylenimine (PolySciences, Warrington, PA, USA). After transfection for 48–72 h, the secreted virus particles in the medium were harvested and filtered, followed by co-incubation with Caco-2 and LoVo cells with approximately 50% confluence in the presence of polybrene. At last, cells were passaged and selected using puromycin.

### 5-ethynyl-2’-deoxyuridine (EdU) assay

In short, 4 × 10^3^ cells were cultured in 96-well plates for 24 h at 37˚C, and then added with EdU solution (20 µM, RiboBio, Guangzhou, China) for another 2 h. After fixture in 4% formaldehyde solution, cells were permeabilized with 0.5% Triton-X-100 and reacted with Apollo reaction cocktail for 30 min. Finally, EdU-positive cells were visualized under a fluorescence microscope after staining with DAPI for 30 min.

### Cell apoptosis and caspase 3 activity assay

After collection and washing, transfected Caco-2 and LoVo cells from each group were mixed in binding buffer. Then, cells were stained with 5 µL Annexin V-FITC (KeyGEN BioTECH, Nanjing, China), followed by mixing with 5 µL PI (KeyGEN BioTECH) for 15 min away from light. The stained cells were assessed using a flow cytometer. In addition, the caspase 3 activity in Caco-2 and LoVo cells was determined based on Caspase 3 Activity Assay Kit (Beyotime).

### Transwell assay

Caco-2 and LoVo cells were plated in Boyden chambers containing 24-transwell plates with 8 μm pore inserts (Chemicon, Temecula, CA, USA). For migration experiments, 5 × 10^4^ cells were introduced into the upper chambers with 200 µL serum-free medium. Meanwhile, the lower counterparts were loaded with 600 µL complete medium. Following a 24-h incubation, migrated cells in lower filters were labeled and counted by a microscope. For invasion assay, 1 × 10^5^ cells were added to the upper chamber pre-coated with matrigel. The subsequent steps are consistent with the above experiment.

### Wound healing assay

In short, Caco-2 and LoVo cells were cultured until 95% confluency. Then, the cell monolayer was scraped linearly to introduce an artificial wound using a 200 µl sterile pipette (record 0 h). Cellular debris removal was done by washing with culture medium. After that, the cells were incubated for 24 h in serum-free fresh medium. At last, the gap size was imaged at 0 h and 24 h using a microscope and Image J software.

### Detection of reactive oxygen species (ROS) and Fe^2+^ ions

For intracellular ROS production, 3 × 10^4^ Caco-2 and LoVo cells in 96-well plates were allowed to culture overnight, followed by staining with 10 µM DCFH-DA (MCE, Monmouth Junction, NJ, USA) at 37˚C. After re-suspending in HBSS, the fluorescence intensity of DCFH-DA was assessed using a flow cytometer. For the intracellular Fe^2+^ ions level, 1 × 10^5^ Caco-2 and LoVo cells were centrifuged. Cell supernatants were collected for the measurement of relative intracellular ferrous iron level according to Total Iron Colorimetric Assay Kit (Elabscience, Houston, TX, USA).

### Immunofluorescence (IF) analyses

In short, Caco-2 and LoVo cells were transfected with sh-NC or sh-TMC5. After fixture in 4% paraformaldehyde, the collected cells were blocked with 5% BSA, followed by incubation with primary antibodies γH2AX (ab81299, 1:40, Abcam), 53BP1 (ab175188, 1:100, Abcam), and secondary antibody. Then, DAPI (Beyotime) was added for nuclear staining. Finally, the fluorescent images were captured using confocal fluorescence microscopy.

### Glycolysis analysis

After transfection for 48 h, the culture medium of Caco-2 and LoVo cells was collected. Then, the levels of glucose consumption and lactate production were determined using Glucose Assay Kit (Sigma-Aldrich) and Lactate Assay Kit (Sigma-Aldrich).

### Tumor xenograft assay

To perform the tumorigenesis assay, 2 × 10^6^ LoVo cells transfected with sh-NC or sh-TMC5 in 100 µL PBS were subcutaneously injected into BALB/C male nude mice (5–6 weeks old, Slaike Jingda Laboratory, Hunan, China) (*n* = 5 per group). During this period, tumor size was measured using a caliper every five days. 30 days after cell inoculation, mice were euthanized for tumor retrieval. Then, the excised tumors were subjected to weighing, and Immunohistochemical (IHC) staining was performed to assess TMC5, ki-67, and heavy chain of ferritin (FTH1)-positive expression. A Mouse xenograft procedure was approved by the Animal Ethics Committee of the Affiliated Hospital of Inner Mongolia Medical University.

### MeRIP

Generally, total RNA from COAD cells was extracted using TRIzol with DNA digestion. Then, RNA samples were sonicated to produce fragments of 100–150 nucleotides. After that, one-ninth of the mRNA was saved as “Input”. The anti-IgG or anti-m6A antibody was pre-incubated with A/G immunomagnetic beads in IP buffer at room temperature for 1 h. Subsequently, the remaining fragment mRNAs were added to the antibody-beads mixture, followed by incubation for 4 h on a rotator at 4˚C. After washing, immunoprecipitated RNAs from each sample were used for RT-qPCR analysis.

### Dual-luciferase reporter assay

To further analyze the interaction between RBM15 and TMC5, this experiment was performed in Caco-2 and LoVo cells. The target sequence of TMC5 possessing the potential m6A site was obtained and inserted into the downstream of pmirGLO luciferase reporter plasmid (Promega, Madison, WI, USA), namely TMC5-wt. Meanwhile, mutations (TMC5-mut) were carried out in the binding sites. After that, a mixture of 800 ng construct and 20 pmol shRNA was transfected into COAD cells in 24-well plates. After 48 h, a dual-luciferase reporter assay system (Promega) was applied for luciferase activities.

### Statistical analysis

The experimental results were processed using GraphPad Prism7 software. Expression association was analyzed using Pearson correlation analysis. Data were shown in the form of mean ± standard deviation (SD). *P* < 0.05 was considered significant. The statistical significance of differences was evaluated based on Student’s *t*-test or one-way analysis of variance (ANOVA) with Tukey’s tests.

## Results

### TMC5 expression was upregulated in COAD

**Fig. 1:**
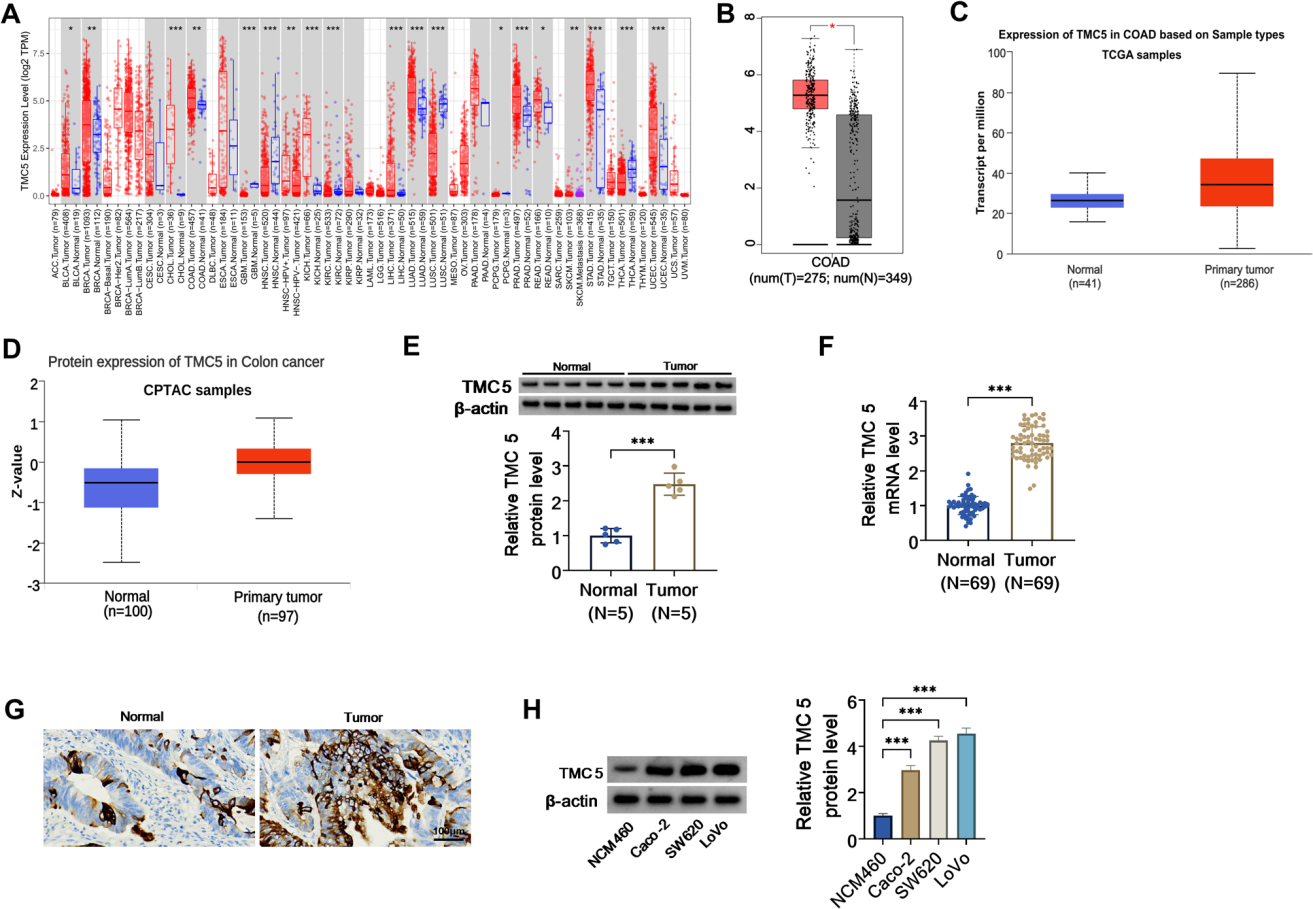
Expression level of TMC5 in COAD. (**A**) Analysis of mRNA expression of TMC5 in different cancers based on TIMER2.0 database. (**B**) GEPIA database (http://gepia.cancer-pku.cn/) exhibited the expression level of TMC5 in COAD samples (*n* = 275) and normal samples (*n* = 349). (**C** and **D**) UALCAN database showed TMC5 mRNA and protein expression based on The Cancer Genome Atlas (TCGA) and Clinical Proteomic Tumor Analysis Consortium (CPTAC) samples. (**E**) TMC5 protein level was determined in 5 normal tissues and 5 COAD tumor tissues using western blot. (**F**) TMC5 mRNA level was detected in 69 normal tissues and 69 COAD tumor tissues using RT-qPCR. (**G**) Immunohistochemical observation of TMC5 expression in normal and COAD tumor tissues. (**H**) Western blot analysis of TMC5 protein level in NCM460 cell line and COAD cell lines (Caco-2, SW620, and LoVo). ***P < 0.001

First of all, to investigate the function of TMC5 on the genesis of human cancers, TIMER database was utilized to analyze the mRNA levels of TMC5 in 61 types of cancers. Data displayed that TMC5 was highly expressed in multiple tumors, including COAD (Fig. 1A). Due to a lack of research on this topic, we further assessed the difference in TMC5 expression between the tumor and normal tissues in COAD. Subsequently, Gene Expression Profiling Interactive Analysis (GEPIA) and UALCAN databases presented that the expression level of TMC5 was significantly elevated in COAD samples compared with normal samples (Fig. 1B and D). Consistent with the above results, an apparent increase in TMC5 was observed in COAD samples relative to normal samples using western blot and RT-qPCR (Fig. 1E and F). Beyond that, IHC staining exhibited that the positive expression rate of TMC5 was also improved in COAD tumor tissues (Fig. 1G). Moreover, western blot assays further verified that TMC5 protein level was clearly enhanced in COAD cell lines (Caco-2, SW620, and LoVo) versus NCM460 cell line (Fig. 1H). Together, these data indicated that dysregulated TMC5 might be involved in COAD progression.

### Knockdown of TMC5 could block COAD cell proliferation, migration, invasion, and induce apoptosis and ferroptosis in vitro

**Fig. 2:**
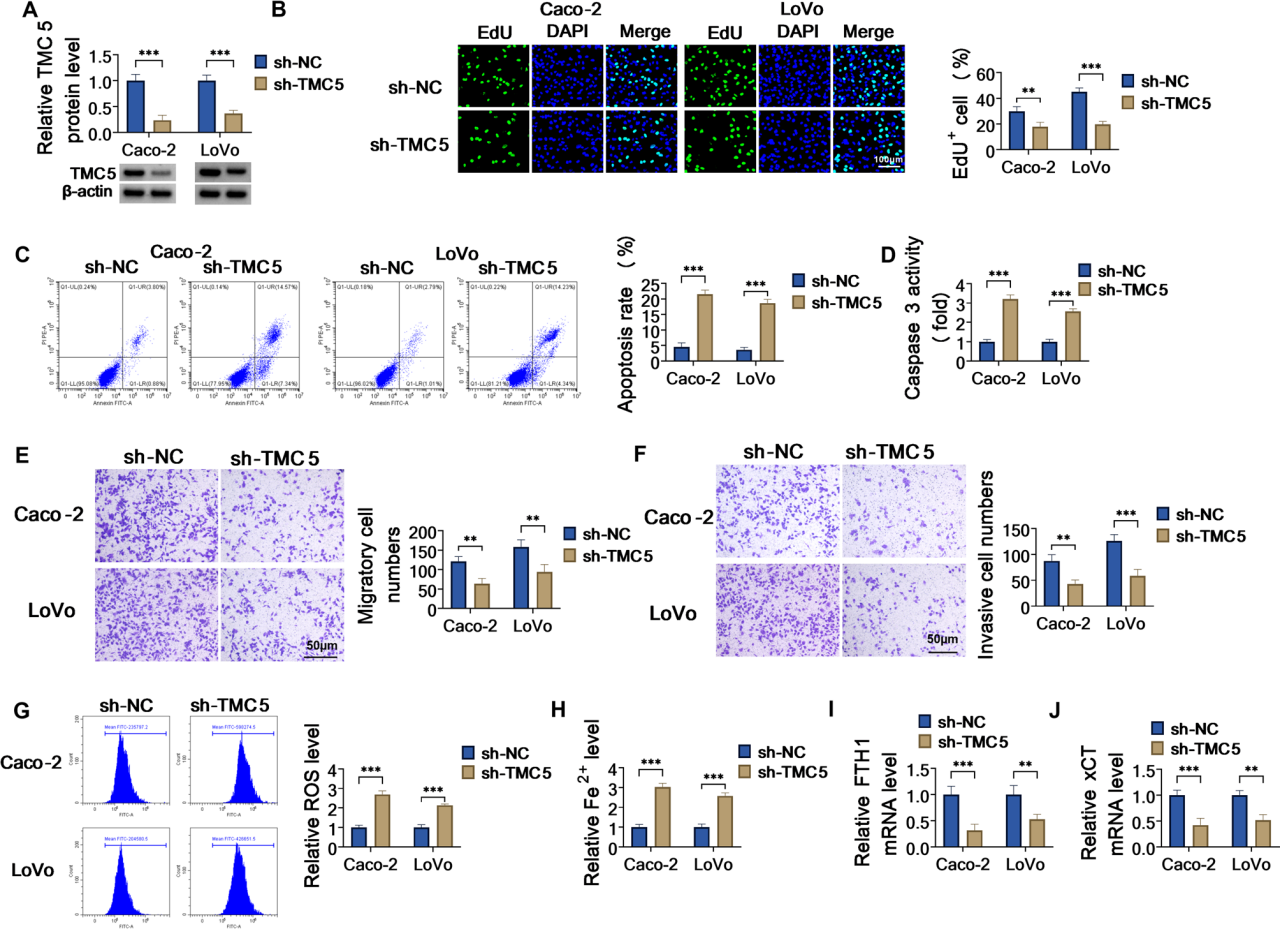
TMC5 downregulation repressed COAD cell development. Caco-2 and LoVo cells were transfected with sh-NC or sh-TMC5. (**A**) TMC5 protein level was determined in transfected Caco-2 and LoVo cells using western blot. (**B**) Cell proliferation was assessed using EdU assay. (**C**) Cell apoptosis was measured using flow cytometry assay. (**D**) Caspase 3 activity was detected using a commercial kit. (**E** and **F**) Cell migration and invasion were examined using Transwell assays. (**G**) ROS level was examined using a commercial kit. (**H**) Iron assay kit was carried out to detect Fe^+^ level in transfected Caco-2 and LoVo cells. (**I** and **J**) FTH1 and xCT mRNA levels were determined using RT-qPCR. **P < 0.01, ***P < 0.001

Then, in vitro loss-of-function analyses were performed to assess the functional role of TMC5 on COAD cell malignant behaviors. At first, western blot analysis exhibited that TMC5 protein level was overtly reduced in sh-TMC5-transfected Caco-2 and LoVo cells in comparison with cells transfected with sh-NC (Fig. 2A), indicating that the interference efficiency is available. Functionally, EdU assay displayed that the deficiency of TMC5 could impair cell proliferation in Caco-2 and LoVo cells (Fig. 2B). Moreover, increased cell apoptosis and enhanced Caspase 3 activity were observed by the downregulation of TMC5 in COAD cells (Fig. 2C and D). Beyond that, Transwell assays presented that the silencing of TMC5 resulted in an obvious decrease in the migration and invasion potential of Caco-2 and LoVo cells (Fig. 2E and F). Consistently, wound healing results also showed that the lack of TMC5 could apparently hinder Caco-2 and LoVo cell migratory ability (Figure S1A). Meanwhile, to further explore the mechanisms by which TMC5 modulates COAD cell proliferation, migration, and invasion, western blot assay was performed to analyze the protein expression. As shown in Figure S1B, TMC5 deficiency in Caco-2 and LoVo cells elicited a corresponding downregulation of mesenchymal markers N-cadherin, Vimentin, and Fibronectin, along with an upregulation of the epithelial marker E-cadherin, implying the repression of TMC5 knockdown on EMT in COAD cells. Ferroptosis is an iron-dependent form of cell death characterized by the iron-dependent accumulation of lipid reactive oxygen species; ferroptosis plays a crucial role in the progression of various cancers, including COAD [32–34]. Next, researchers detected the role of TMC5 for ferroptosis. In terms of ferroptosis, increasing intracellular Fe^+^ and ROS in Caco-2 and LoVo cells were found after sh-TMC5 transfection (Fig. 2G and H). Simultaneously, two key regulators (FTH1 and xCT) of ferroptosis were further determined to assess the effects of TMC5 on ferroptosis using RT-qPCR. As displayed in Fig. 2I and J, the depletion of TMC5 could remarkably decrease FTH1 and xCT mRNA levels in Caco-2 and LoVo cells. In addition, we further analyzed the effects of TMC5 downregulation on the DNA repair pathway by detecting indicators for DNA double-strand break (DSB) damage, such as 53BP1 and γH2AX. Then, immunofluorescence assay exhibited that the downregulation of TMC5 could obviously enhance the proportion of 53BP1 and γH2AX-positive cells in Caco-2 and LoVo cells (Figure S2A). Meanwhile, western blot results showed that RAD51 (a key protein involved in DSBs and homologous recombination repair) protein level was apparently blocked in COAD cells by TMC5 silencing (Figure S2B). These data implied that TMC5 knockdown promotes the DNA damage and represses the DNA repair pathway in COAD cells. Considering that glycolysis is the primary pathway for tumor cells to obtain energy, and that increased glucose uptake and lactate production are important characteristics of glycolysis activation, we examined the effects of TMC5 downregulation on glucose uptake and lactate production. Results exhibited that the lack of TMC5 could markedly repress glucose consumption and lactate production in Caco-2 and LoVo cells (Figure S3), suggesting the repression of TMC5 downregulation on the glycolysis in COAD cells. Collectively, these results indicated that the lack of TMC5 could suppress COAD cell malignant behaviors in vitro.

### Silencing of TMC5 repressed COAD cell growth in vivo

**Fig. 3:**
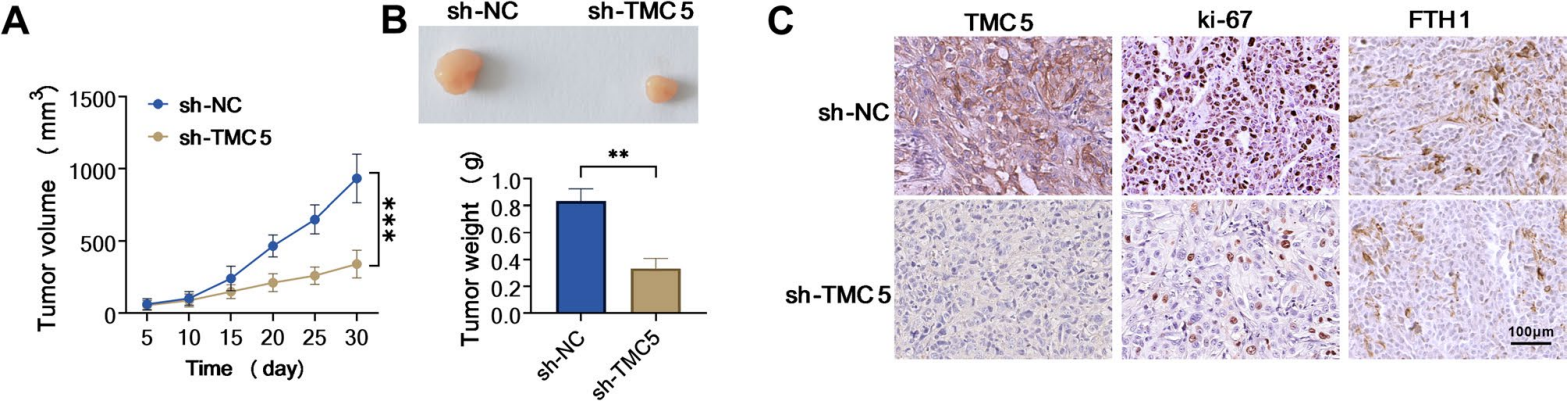
Inhibiting TMC5 impaired COAD cell growth in vivo. sh-NC or sh-TMC5-transfected LoVo cells were subcutaneously injected into mice. (**A** and **B**) Tumor volume and tumor weight were examined. (**C**) IHC staining was used to assess the positive expression of TMC5, ki-67, and FTH1 in xenografted tumor samples. **P < 0.01, ***P < 0.001

Furthermore, to validate the functional effect of TMC5 on COAD cell malignancy in vivo, a xenograft tumor mouse model was established. As shown in Fig. 3A and B, tumor size and weight dropped in the presence of TMC5 deficiency, indicating that the downregulation of TMC5 could efficiently hinder the cell growth of COAD in vivo. Apart from that, IHC staining observed that the positive expression rates of TMC5, ki-67, and FTH1 were clearly dampened in the tissue samples from the sh-TMC5 group than the sh-NC group (Fig. 3C). Therefore, it is concluded that blocking TMC5 could inhibit COAD cell growth *in vivo.*

### RBM15 increases m6A modification and expression of TMC5 mRNA

**Fig. 4:**
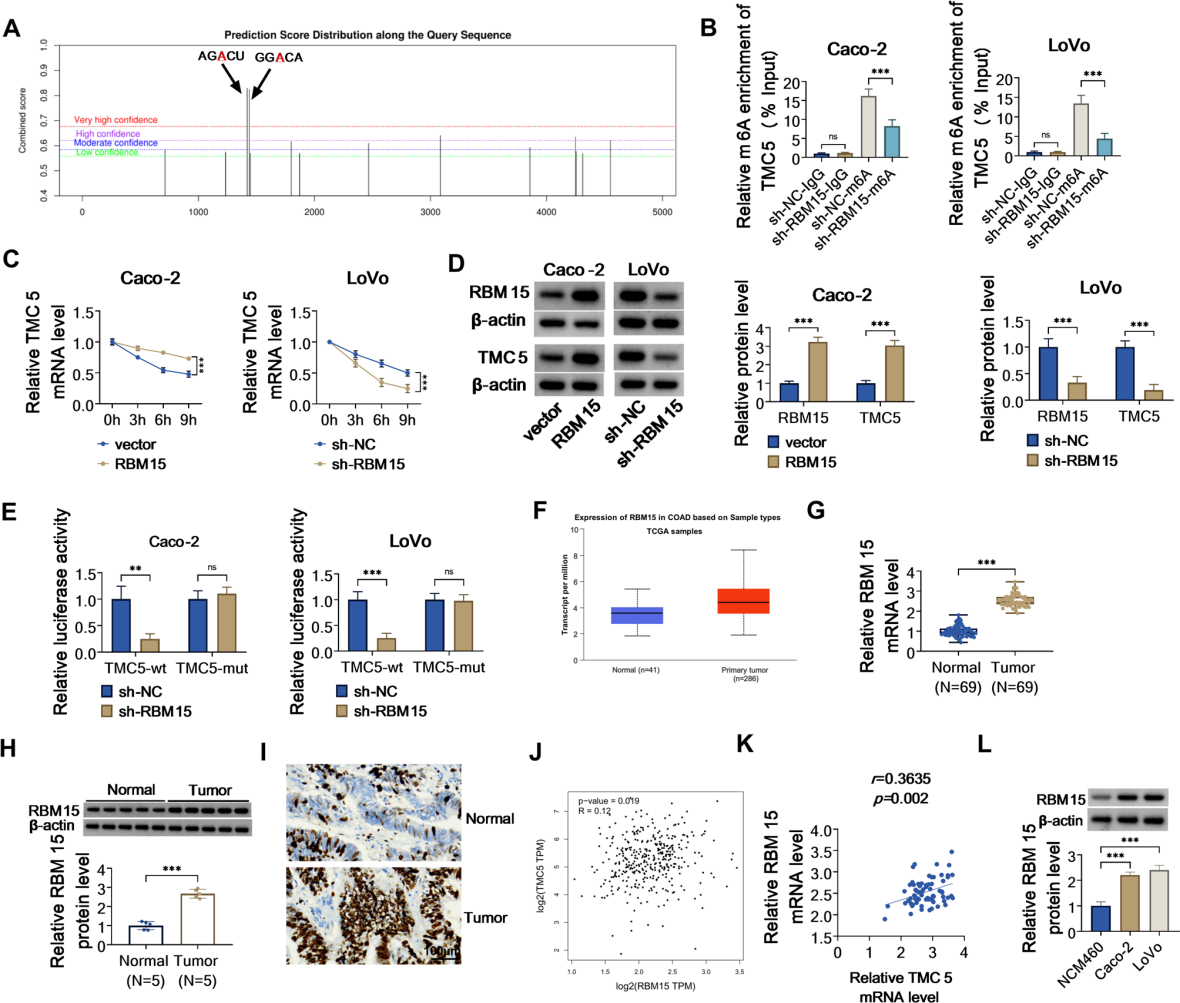
RBM15 modulated m6A enrichment and stability of TMC5 mRNA. (**A**) The online tool SRAMP was used to predict m6A sites of TMC5. (**B**) Alterations in the m6A methylation level of TMC5 after inhibition of RBM15 were analyzed by MeRIP-qPCR assay. (**C**) Influences of RBM15 overexpression or downregulation on TMC5 mRNA stability after Actinomycin D treatment was measured using RT-qPCR in Caco-2 and LoVo cells. (**D**) RBM15 and TMC5 protein levels were determined in Caco-2 and LoVo cells transfected with vector, RBM15, sh-NC, or sh-RBM15 using western blot. (**E**) Their interaction was confirmed using dual-luciferase reporter assay in Caco-2 and LoVo cells. (**F**) UALCAN database presented the expression level of RBM15 in COAD samples (*n* = 286) and normal samples (*n* = 41) based on sample types TCGA samples. (**G**) RT-qPCR analysis of RBM15 mRNA level in 69 normal tissues and 69 COAD tumor tissues. (**H**) Western blot analysis of RBM15 protein level in 5 normal tissues and 5 COAD tumor tissues. (**I**) IHC staining analyzed the positive expression rate RBM15 in normal tissues and COAD tumor tissues. (**J**) GEPIA database exhibited the association between RBM15 and TMC5 expression in COAD samples. (**K**) Pearson correlation analysis was applied to evaluate the expression correlation between RBM15 and TMC5 in COAD tissues. (**L**) RBM15 protein level was detected in NCM460 cell line and COAD cell lines (Caco-2 and LoVo) using western blot. **P < 0.01, ***P < 0.001

According to the sequence-based RNA adenosine methylation site predictor (SRAMP) website, there are m6A modification sites on the TMC5 mRNA (Fig. 4A). To enhance the understanding of the role of m6A modification in the regulation of TMC5 expression, MeRIP-qPCR assay was conducted in Caco-2 and LoVo cells. As shown in Fig. 4B, inhibition of RBM15 significantly decreased the m6A level of TMC5 mRNAs. When cells were treated with Actinomycin D to block the synthesis of mRNA, TMC5 mRNA stability was prolonged upon RBM15 overexpressing in Caco-2 cells, and its stability was reduced due to RBM15 downregulation in LoVo cells (Fig. 4C). Furthermore, we found that the upregulation of RBM15 could apparently improve TMC5 protein level in Caco-2 cells, whereas RBM15 disruption could markedly decline TMC5 protein level in LoVo cells (Fig. 4D). Apart from that, the luciferase activity of TMC5-wt, rather than the mutant group, was significantly reduced by the deficiency of RBM15 in Caco-2 and LoVo cells (Fig. 4E). Consistent with TMC5 expression, UALCAN databases presented that the expression level of RBM15 was also overtly elevated in COAD samples compared with normal samples (Fig. 4F). Moreover, we further validated that RBM15 content was evidently upregulated in COAD tumor tissues versus normal tissues (Fig. 4G and H). As expected, IHC staining found that the positive expression rate of RBM15 was distinctly elevated in COAD samples (Fig. 4I). In addition, the GEPIA database exhibited that RBM15 expression was positively correlated with TMC5 in COAD samples (Fig. 4J). Consistently, we found that RBM15 has a positive association with TMC5 expression in COAD clinical tissues (Fig. 4K). Apart from that, western blot analysis further validated that RBM15 was highly expressed in COAD cell lines (Caco-2 and LoVo) relative to the NCM460 cell line (Fig. 4L). Taken together, our findings suggested RBM15 could promote TMC5 expression by regulating the RNA m6A modification.

### Inhibition of RBM15 could impair COAD cell malignant behaviors by regulating TMC5

**Fig. 5:**
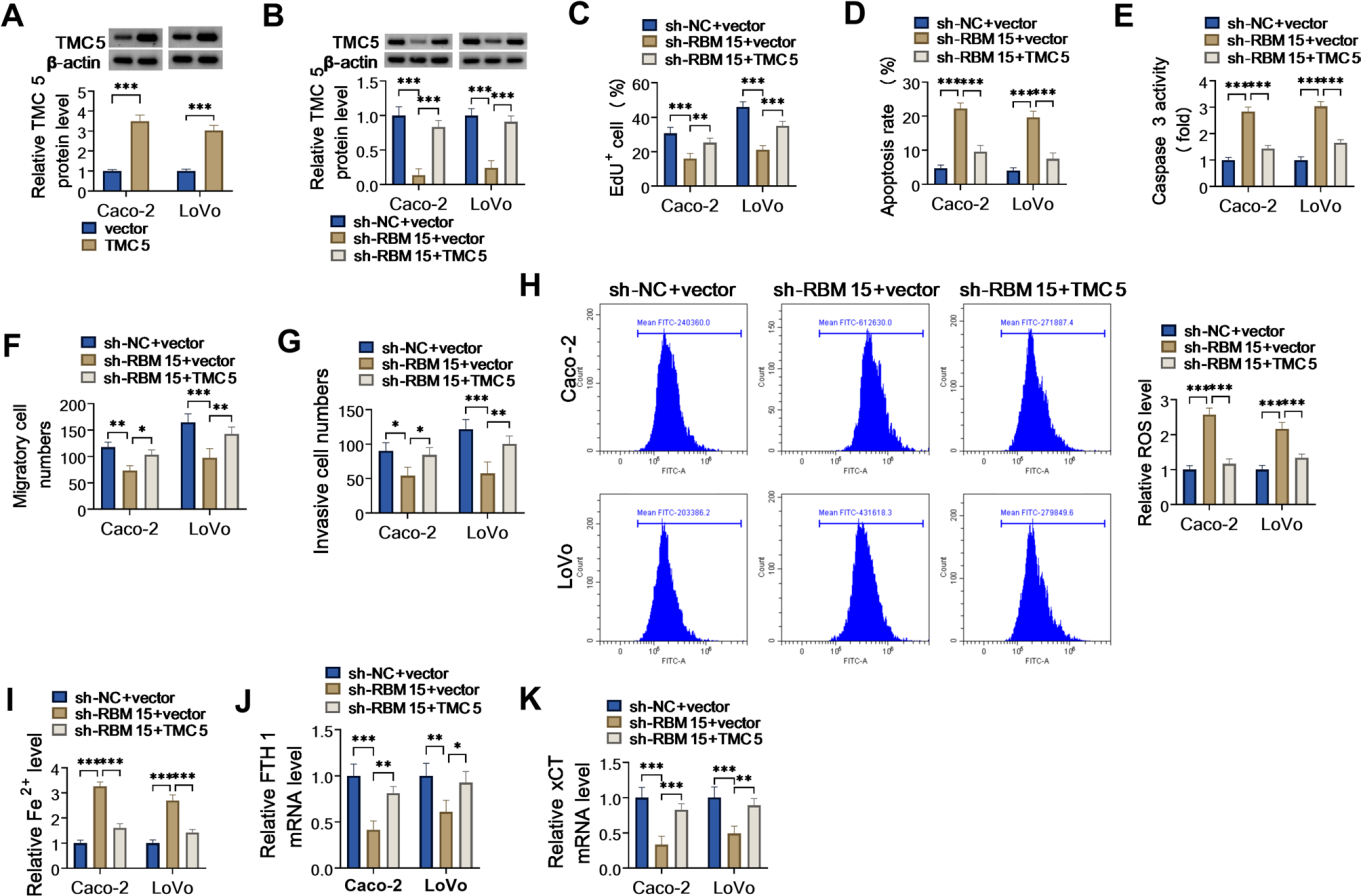
RBM15/TMC5 regulated COAD cell malignant behaviors. (**A**) Western blot analysis of TMC5 protein level in Caco-2 and LoVo cells transfected with vector or TMC5. (**B-K**) Caco-2 and LoVo cells were transfected with sh-NC + vector, sh-RBM15 + vector, or sh-RBM15 + TMC5. (**B**) TMC5 protein level was monitored by western blot. (**C** and **D**) Cell proliferation and apoptosis were tested using EdU and flow cytometry assays. (**E**) Caspase 3 activity was examined using a commercial kit. (**F** and **G**) Cell migration and invasion were measured using Transwell assays. (**H** and **I**) ROS level and Fe^+^ level were determined by special kits. (**J** and **K**) FTH1 and xCT mRNA levels were examined using RT-qPCR. *P < 0.05, **P < 0.01, ***P < 0.001

Additionally, rescue experiments were performed to further explore the effects of RBM15 and TMC5 on COAD cell development. The overexpression efficiency of TMC5 in Caco-2 and LoVo cells was detected and shown in Fig. 5A. Subsequently, western blot results displayed that the co-transfection of TMC5 could remarkably counteract the repression of sh-RBM15 on the TMC5 protein level in Caco-2 and LoVo cells (Fig. 5B). After that, EdU analysis disclosed that the downregulation of RBM15 could strikingly constrain cell proliferation ability in Caco-2 and LoVo cells, which was partly abolished by TMC5 overexpression (Fig. 5C). Moreover, RBM15 deficiency-mediated enhancement in apoptotic rate and caspase-3 activity in COAD cells was notably relieved after co-transfection with TMC5 (Fig. 5D and E). What’s more, the lack of RBM15 elicited an obvious decrease in COAD cell migration and invasion, while these effects were greatly abrogated through TMC5 upregulation (Fig. 5F and G). Besides, the forced expression of TMC5 prominently attenuated RBM15 depletion-triggered ferroptosis promotion in Caco-2 and LoVo cells, accompanied by reduced ROS and Fe^+^ levels (Fig. 5H and I), and increased FTH1 and xCT expression (Fig. 5J and K). All of these findings implied that TMC5 upregulation partially reversed the repression of RBM15 knockdown on COAD cell development.

## Discussion

In recent years, the TMC protein family has attracted extensive attention for their vital roles in various cancers and clinical value [17, 35]. In fact, some studies have indicated that alteration in members of the TMC family is closely associated with the development and progression of human tumors [36, 37]. As a member of the TMC family, TMC5 expression level was strongly linked to shorter overall survival or more advanced stages in most cancers [38, 39]. Furthermore, the altered status of TMC5, containing methylation and mutation, is also correlated with tumor prognosis [20]. The upregulation of TMC5 in cancerous epithelial cells was identified in pancreatic adenocarcinoma [40]. It has been reported that the lack of TMC5 could significantly hinder prostate cancer cell proliferation and improve cell sensitivity to 5-Fluorouracil [21]. Notably, proteomic and phosphoproteomic analysis identified TMC5 as a distinctly dysregulated differential protein in colorectal cancer [22]. Therefore, it is reasonable to hypothesize that TMC5 might be related to carcinogenesis and progression in COAD.

Herein, database analysis revealed that TMC5 was clearly increased in COAD. Consistently, TMC5 content was also enhanced in COAD. Consistent with roles in other cancers, TMC5 silencing could dampen COAD cell proliferation, metastasis, and glycolysis. Similarly, TMC5 deficiency hindered COAD cell growth in vivo. As a non-apoptotic form of regulated cell death, ferroptosis has been reported as an innate tumor suppressor mechanism and is involved in many biological processes of tumors [41, 42]. It is initiated by intracellular iron, resulting in lipid peroxidation accumulation, thereby promoting cell death [43]. Furthermore, the SLC7A11 (xCT)/GPX4 axis delivers antioxidant effects by diminishing lipid peroxide accumulation, thus inhibiting ferroptosis [44]. Meanwhile, FTH1, a key modulator in regulating iron metabolism in ferroptosis, could impair the ferroptosis activation pathways by improving iron storage and reducing cellular Fe^2+^ levels [45]. Hence, we further explored the effects of TMC5 on ferroptosis in COAD cells. Herein, our data confirmed that TMC5 knockdown could induce ROS and Fe^2+^ levels and repress xCT and FTH1 expression in COAD cells, validating the promotion of TMC5 downregulation on ferroptosis. In addition, previous studies have indicated that ROS are known to cause various types of DNA damage and affect the DNA damage response, thereby regulating cell proliferation and apoptosis [46, 47]. Herein, the current work verified that the lack of TMC5 could promote the DNA damage of COAD cells. These findings implied that TMC5 might be a carcinogenic factor in COAD for the first time.

Regarding the molecular mechanism, numerous researchers have stated that m6A modification could alter gene expression by affecting mRNA stability and splicing at the cellular level during tumor development [48, 49]. As a universal regulator of m6A RNA methylation, RBM15 has been reported to be dysregulated in various human cancers, including COAD [29]. Furthermore, previous studies have demonstrated that elevated RBM15 could accelerate colorectal cancer cell growth and metastasis through m6A modification of MyD88 and KLF1 mRNA [30, 31]. In the present work, TMC5 was identified as a downstream target of RBM15, and RBM15 could sustain mRNA stability and TMC5 expression in COAD cells. More importantly, lacking RBM15-triggered COAD cell growth inhibition and ferroptosis promotion was, at least in part, overturned through TMC5 overexpression. On the whole, these observations preliminarily corroborated the RBM15/TMC5 regulatory mechanism during COAD progression.

## Conclusion

Taken together, our findings provided compelling evidence that RBM15, a key m6A methyltransferase, could expedite COAD cell growth and repress ferroptosis by increasing TMC5 expression. These results provide a new target for COAD diagnosis in the future.

## Supplementary Information

Below is the link to the electronic supplementary material.

Supplementary Material 1

Supplementary Material 2

## Data Availability

No datasets were generated or analysed during the current study.
