# Supplementary material for: RBM15 promotes COAD progression by regulating the m6A modification of TMC5
Source: Hereditas. 2025 Aug 29;162:177. doi: 10.1186/s41065-025-00530-4 (PMC12395726; doi:10.1186/s41065-025-00530-4)
Supplement: Supplementary file 1 — Supplementary Material 1 [file 41065_2025_530_MOESM1_ESM.pptx]

## Slide 1
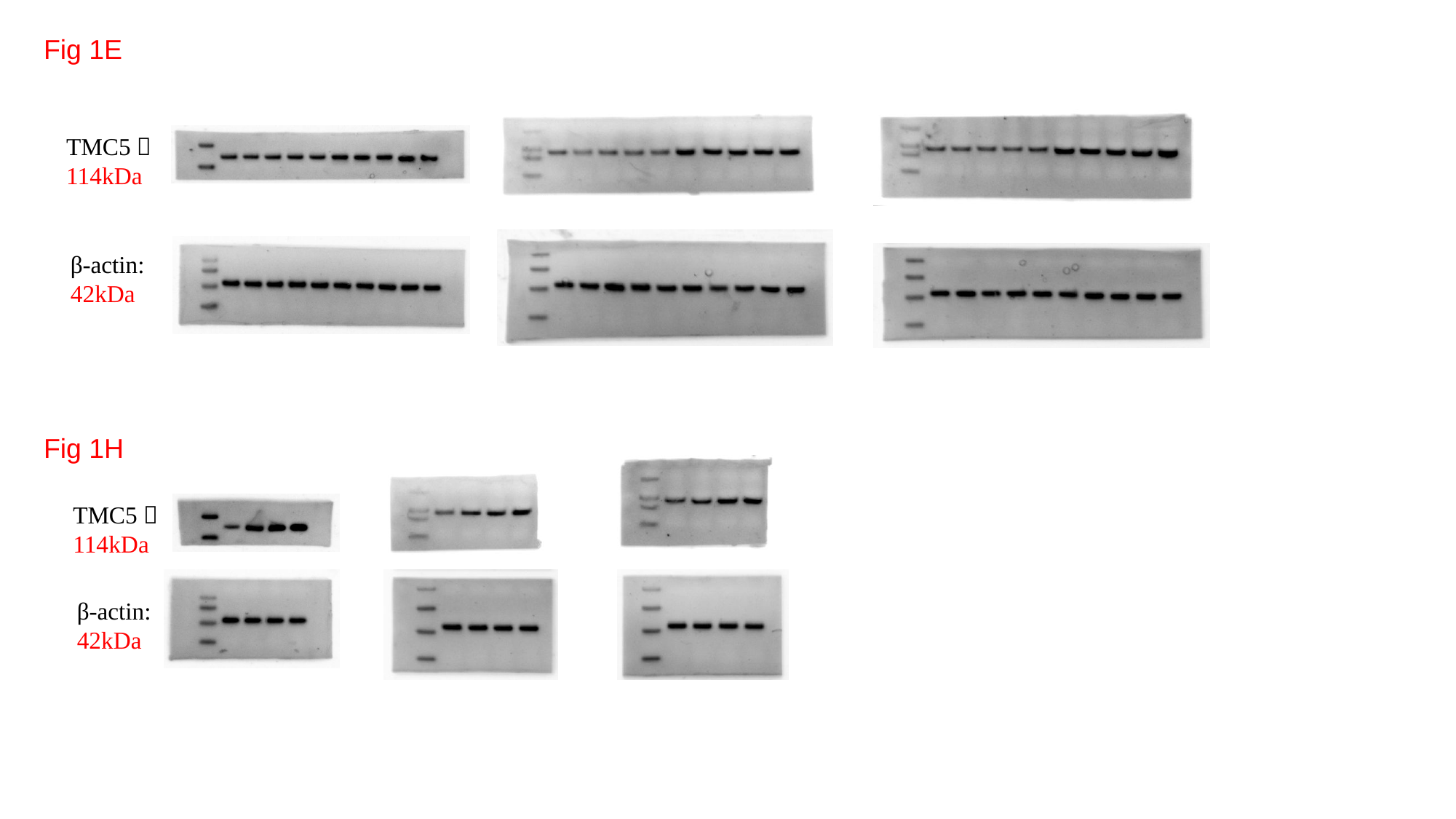

Fig 1E
TMC5：
114kDa
β-actin:
42kDa
Fig 1H
TMC5：
114kDa
β-actin:
42kDa

## Slide 2
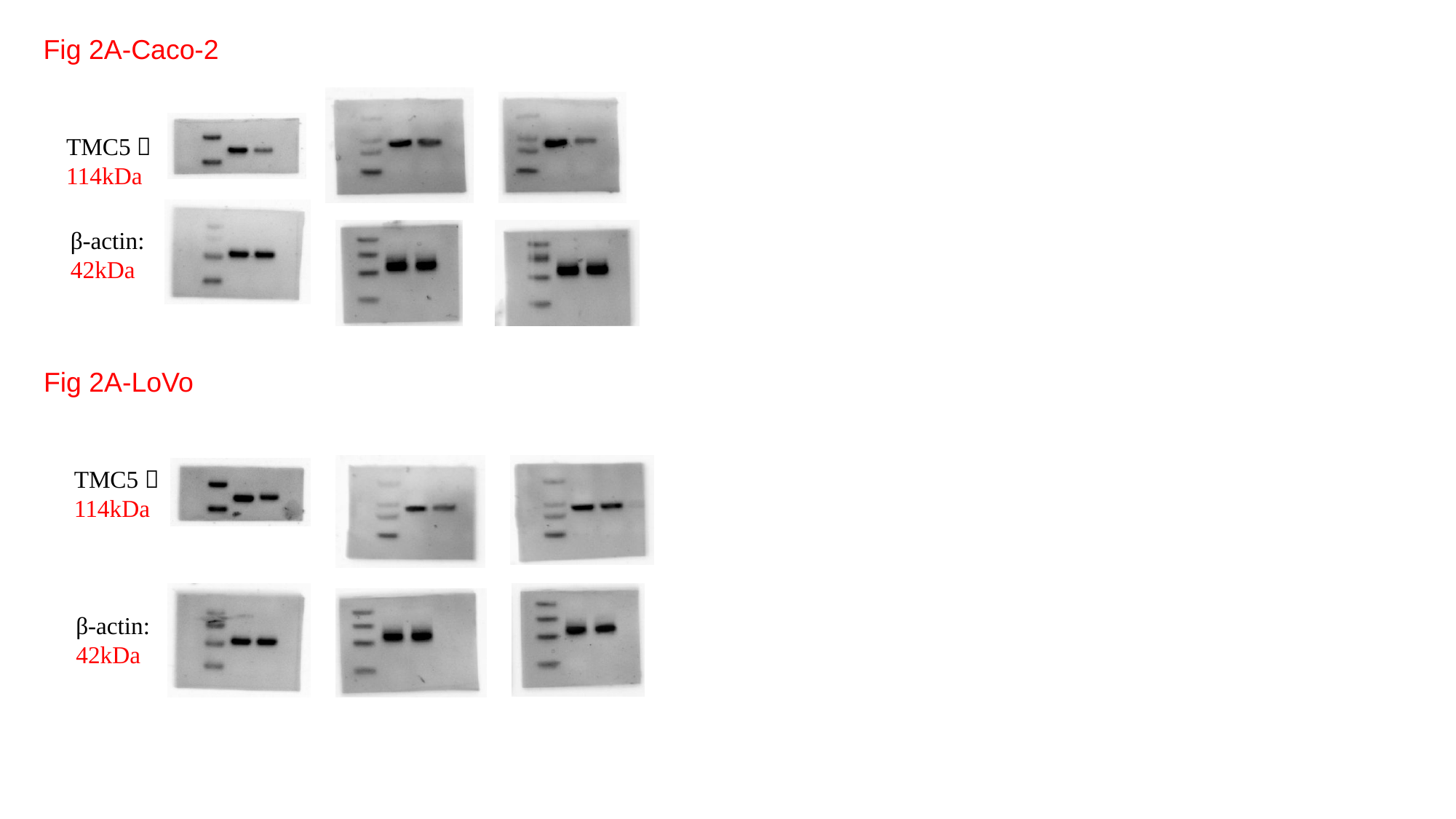

Fig 2A-Caco-2
TMC5：
114kDa
β-actin:
42kDa
Fig 2A-LoVo
TMC5：
114kDa
β-actin:
42kDa

## Slide 3
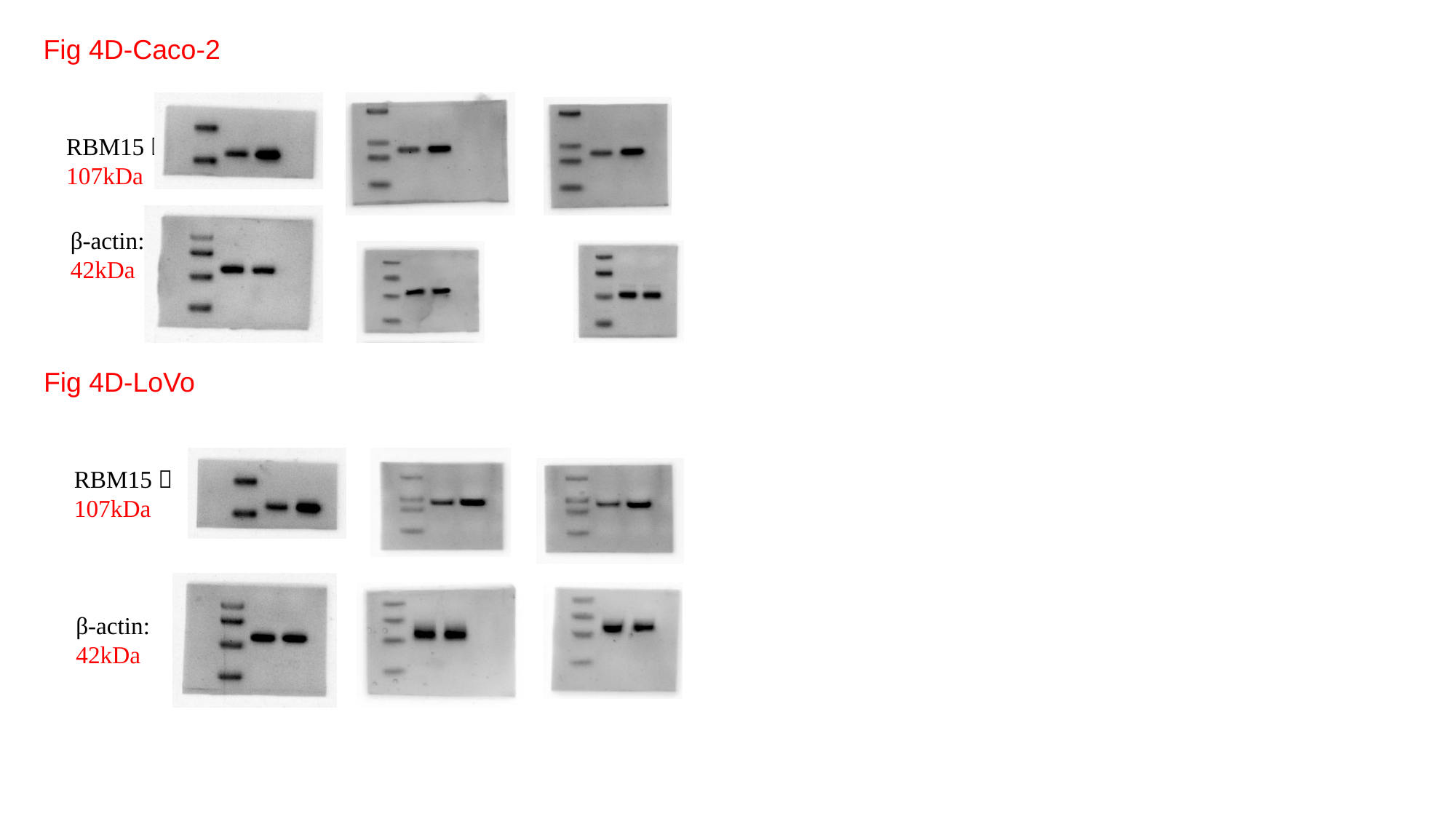

Fig 4D-Caco-2
RBM15：
107kDa
β-actin:
42kDa
Fig 4D-LoVo
RBM15：
107kDa
β-actin:
42kDa

## Slide 4
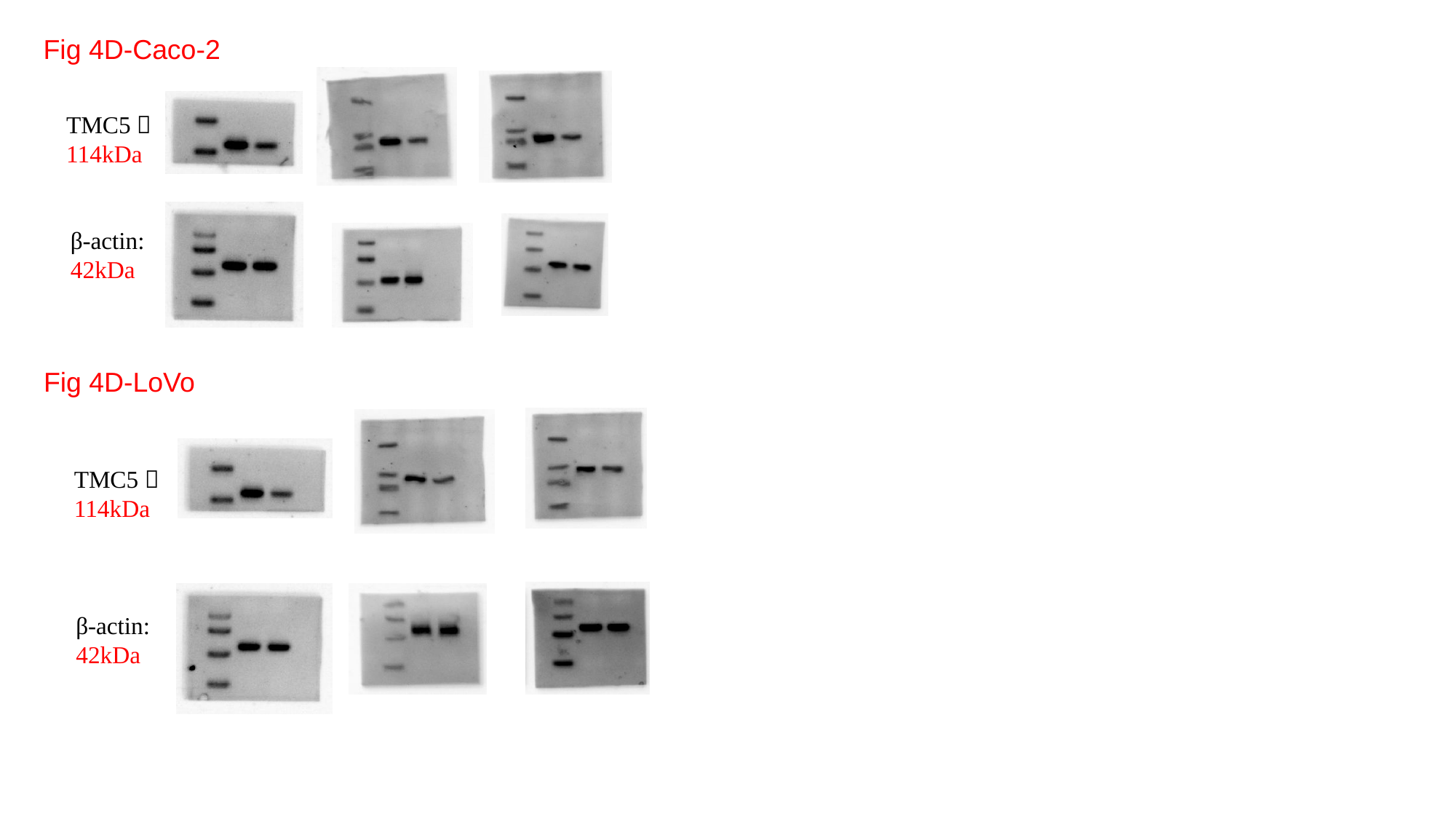

Fig 4D-Caco-2
TMC5：
114kDa
β-actin:
42kDa
Fig 4D-LoVo
TMC5：
114kDa
β-actin:
42kDa

## Slide 5
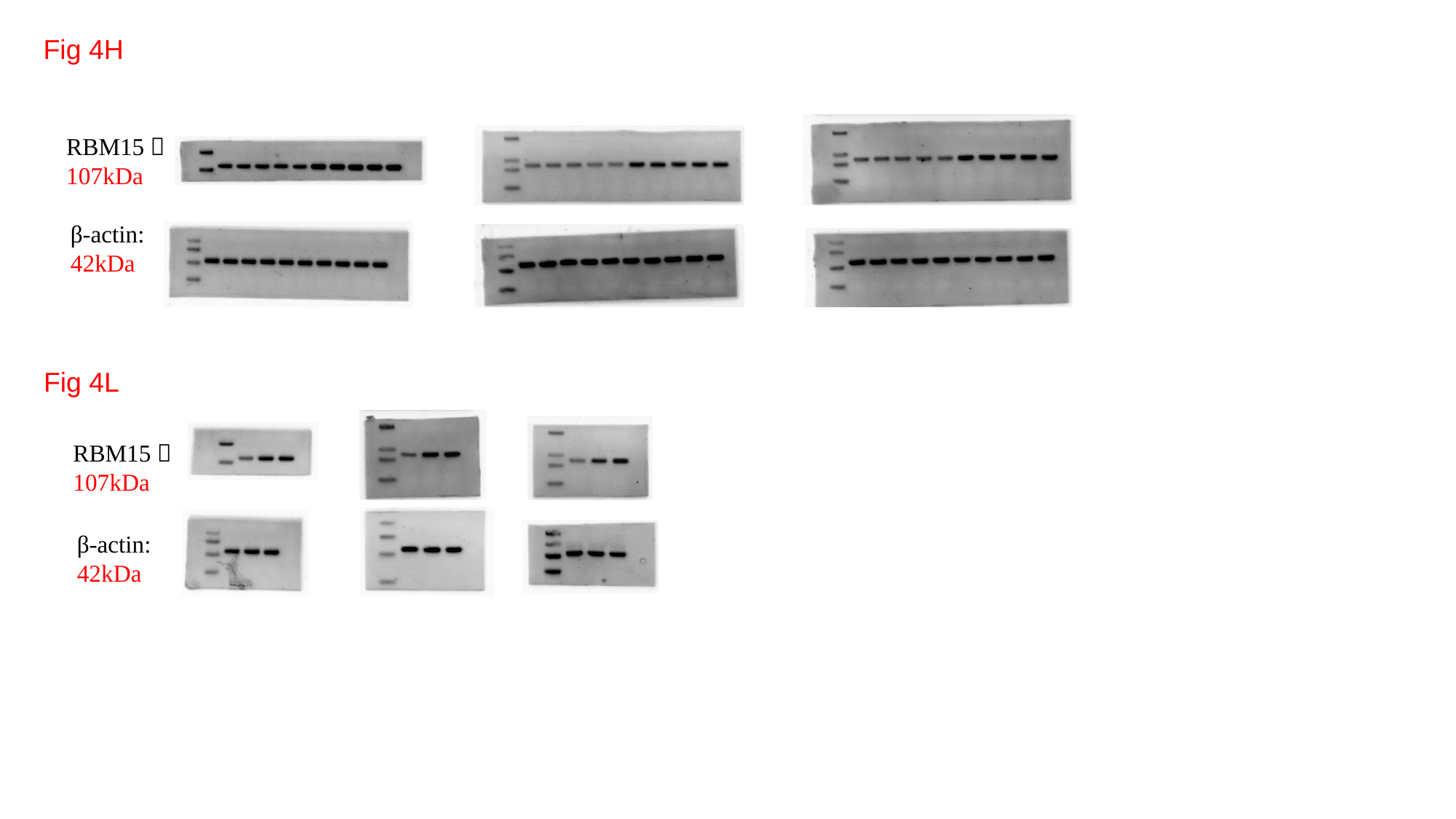

Fig 4H
RBM15：
107kDa
β-actin:
42kDa
Fig 4L
RBM15：
107kDa
β-actin:
42kDa

## Slide 6
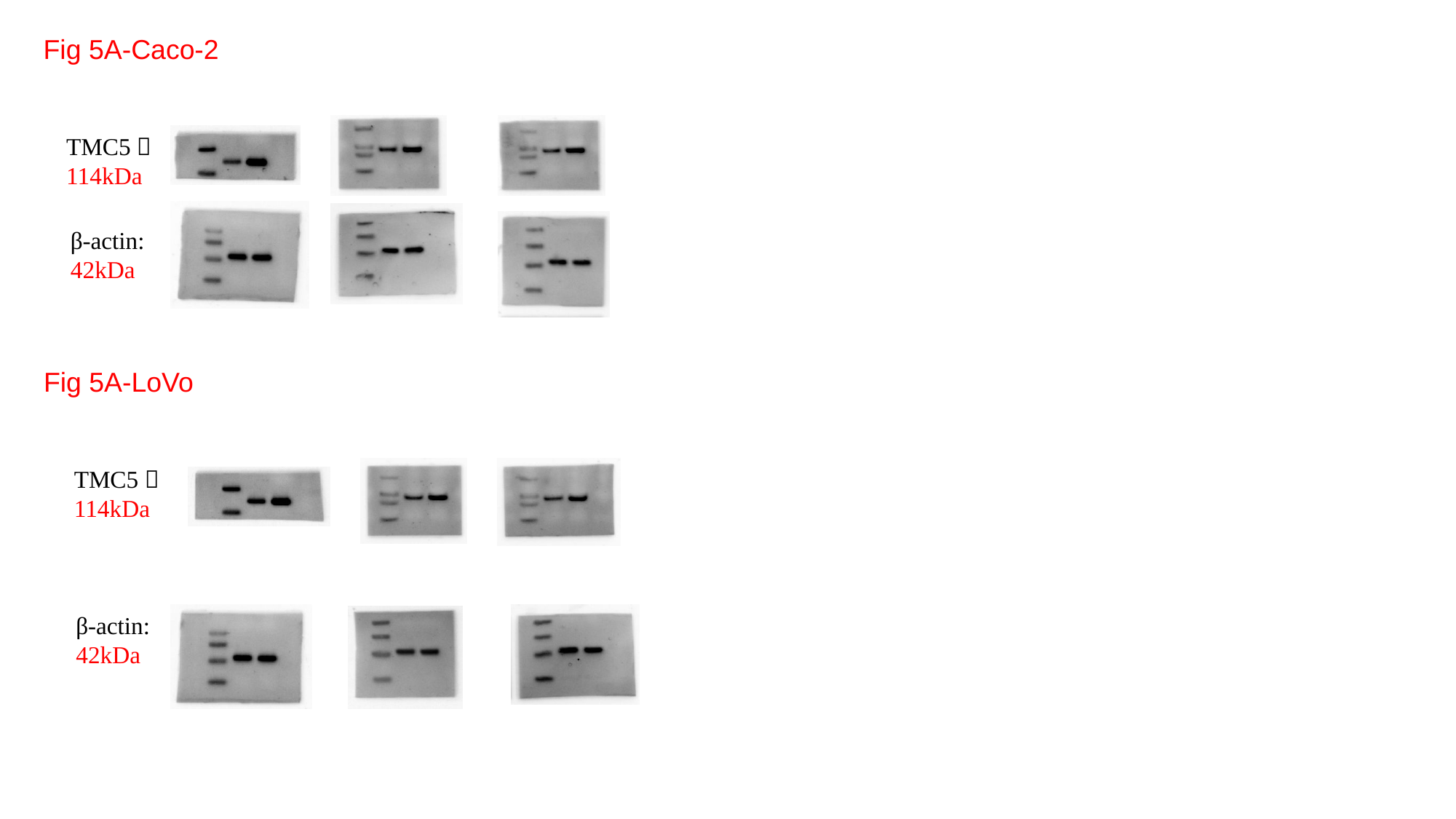

Fig 5A-Caco-2
TMC5：
114kDa
β-actin:
42kDa
Fig 5A-LoVo
TMC5：
114kDa
β-actin:
42kDa

## Slide 7
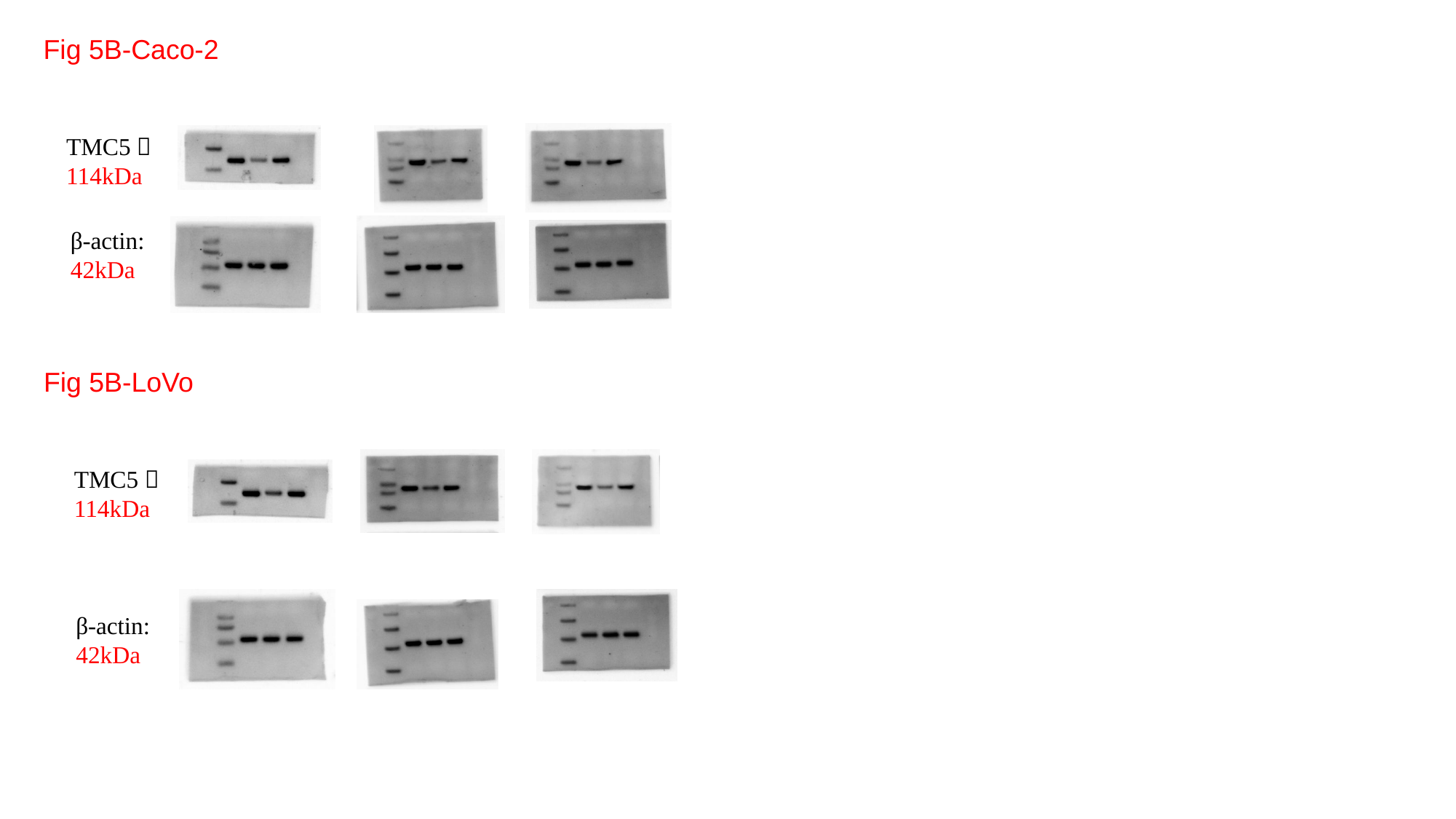

Fig 5B-Caco-2
TMC5：
114kDa
β-actin:
42kDa
Fig 5B-LoVo
TMC5：
114kDa
β-actin:
42kDa
