# Supplementary material for: RBM15 promotes COAD progression by regulating the m6A modification of TMC5
Source: Hereditas. 2025 Aug 29;162:177. doi: 10.1186/s41065-025-00530-4 (PMC12395726; doi:10.1186/s41065-025-00530-4)
Supplement: Supplementary file 2 — Supplementary Material 2 [file 41065_2025_530_MOESM2_ESM.docx]

**
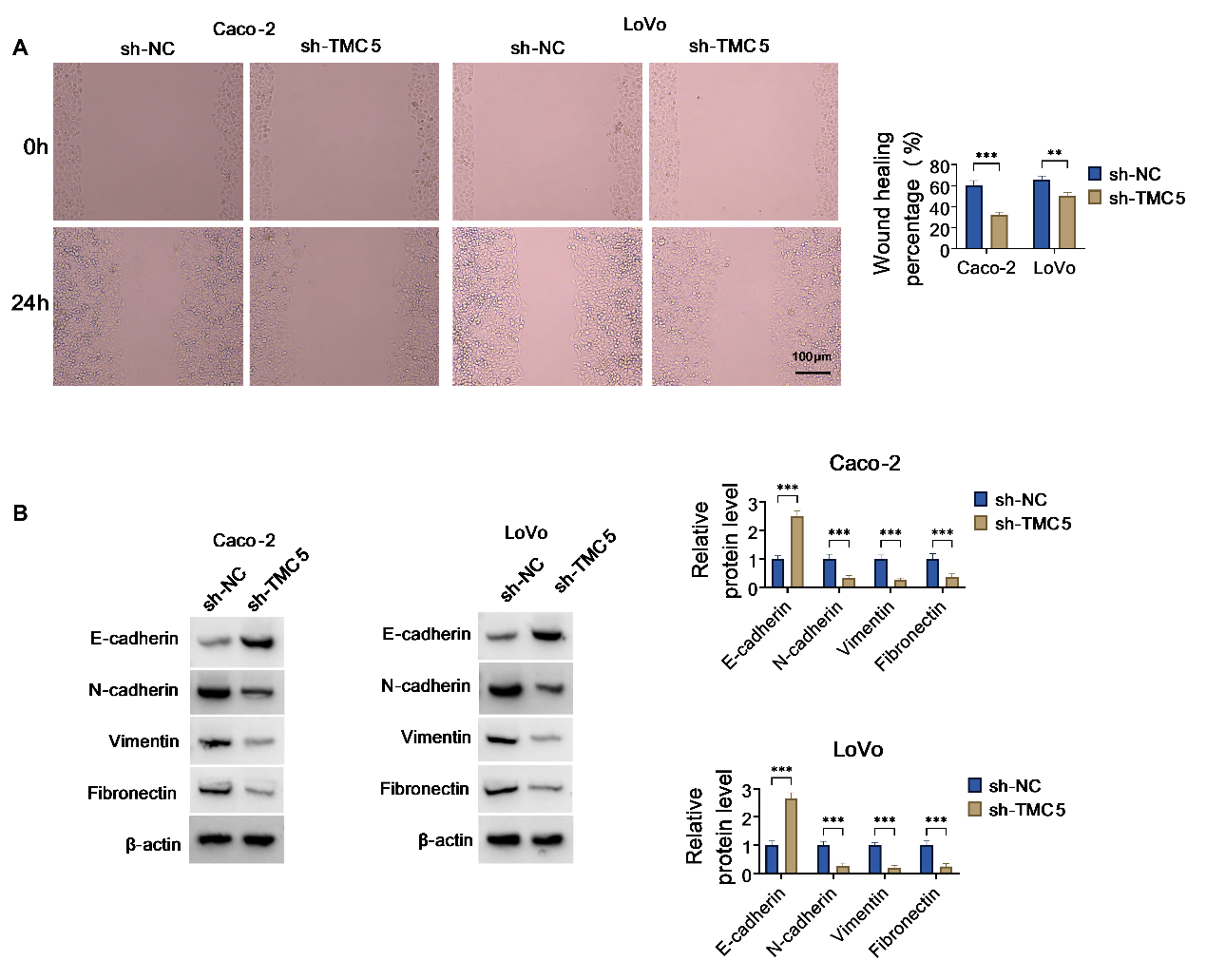
**

**Figure S1** **TMC5 knockdown blocked** **COAD cell migratory ability and EMT.** Caco-2 and LoVo cells were transfected with sh-NC or sh-TMC5. (A) Cell migration was measured using wound healing assay. (B) E-cadherin, N-cadherin, Vimentin, and Fibronectin protein levels were determined using western blot. ***P* <0.01, ****P* <0.001.

**
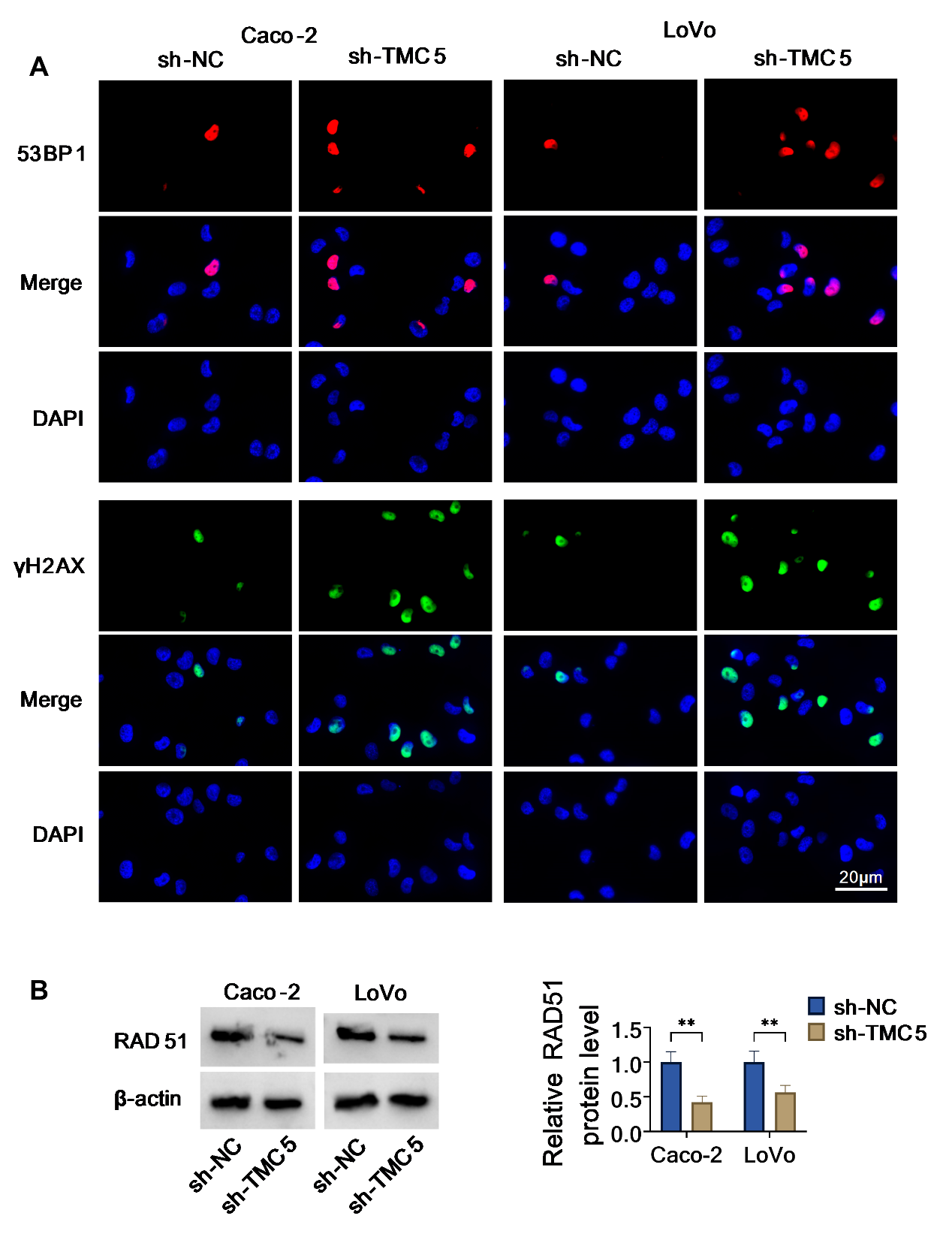
**

**Figure S2 The effects of TMC5 knockdown on** **DNA repair pathways in COAD cells.** Caco-2 and LoVo cells were transfected with sh-NC or sh-TMC5. (A) Immunofluorescence assay was performed to detect the 53BP1 and γH2AX foci in transfected Caco-2 and LoVo cells. (B) RAD51 protein level was determined using western blot. ***P* <0.01.

**
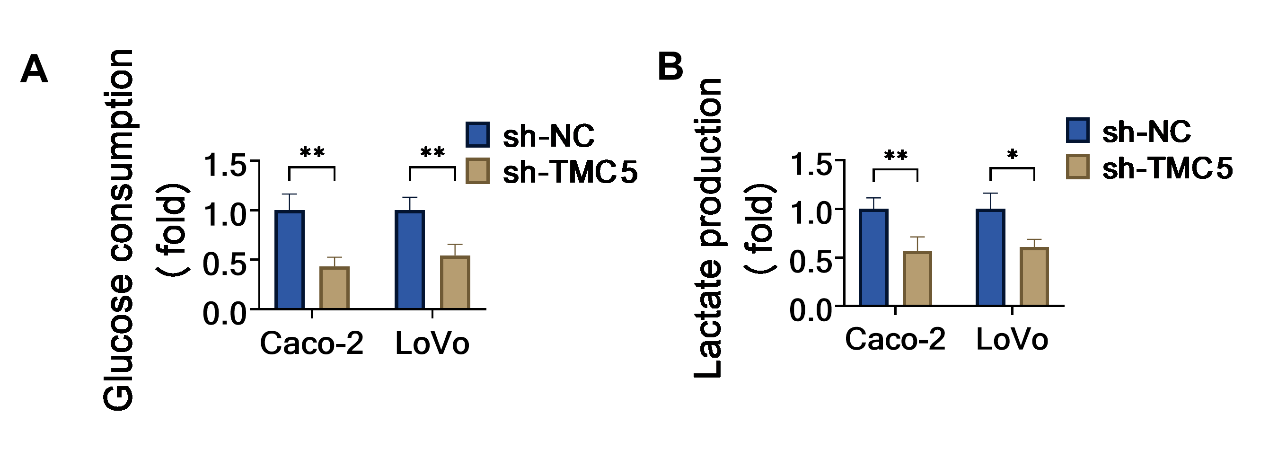
**

**Figure S3 The effects of TMC5 silencing on COAD cell** **metabolism.** Caco-2 and LoVo cells were transfected with sh-NC or sh-TMC5. (A and B) Glucose consumption and lactate production were detected using commercial kits. **P* <0.05, ***P* <0.01.
